# Phenotypic and genotypic background underlying variations in fatty acid composition and sensory parameters in European bovine breeds

**DOI:** 10.1186/2049-1891-5-20

**Published:** 2014-04-15

**Authors:** Natalia Sevane, Hubert Levéziel, Geoffrey R Nute, Carlos Sañudo, Alessio Valentini, John Williams, Susana Dunner

**Affiliations:** 1 Departamento de Producción Animal, Facultad de Veterinaria, Universidad Complutense de Madrid, Madrid, Spain; 2 INRA, UMR 1061, F-87000 Limoges, France; 3 Université de Limoges, UMR 1061, F-87000 Limoges, France; 4 Division of Farm Animal Science, University of Bristol, Bristol BS40 5DU, UK; 5 Departimento de Producción Animal y Ciencia de los Alimentos, Universidad de Zaragoza, 50013 Zaragoza, Spain; 6 Dipartimento di Produzioni Animali, Università della Tuscia, via De Lellis, 01100 Viterbo, Italy; 7 Parco Tecnologico Padano, Via Einstein, Polo Universitario, 26900 Lodi, Italy

**Keywords:** Bos taurus, Beef, Fatty acids, Omega-3, Genotype assisted selection

## Abstract

**Background:**

Consuming moderate amounts of lean red meat as part of a balanced diet valuably contributes to intakes of essential nutrients. In this study, we merged phenotypic and genotypic information to characterize the variation in lipid profile and sensory parameters and to represent the diversity among 15 cattle populations. Correlations between fat content, organoleptic characteristics and lipid profiles were also investigated.

**Methods:**

A sample of 436 largely unrelated purebred bulls belonging to 15 breeds and reared under comparable management conditions was analyzed. Phenotypic data -including fatness score, fat percentage, individual fatty acids (FA) profiles and sensory panel tests- and genotypic information from 11 polymorphisms was used.

**Results:**

The correlation coefficients between muscle total lipid measurements and absolute vs. relative amounts of polyunsaturated FA (PUFA) were in opposite directions. Increasing carcass fat leads to an increasing amount of FAs in triglycerides, but at the same time the relative amount of PUFAs is decreasing, which is in concordance with the negative correlation obtained here between the percentage of PUFA and fat measurements, as well as the weaker correlation between total phospholipids and total lipid muscle content compared with neutral lipids. Concerning organoleptic characteristics, a negative correlation between flavour scores and the percentage of total PUFA, particularly to n-6 fraction, was found. The correlation between juiciness and texture is higher than with flavour scores. The distribution of SNPs plotted by principal components analysis (PCA) mainly reflects their known trait associations, although influenced by their specific breed allele frequencies.

**Conclusions:**

The results presented here help to understand the phenotypic and genotypic background underlying variations in FA composition and sensory parameters between breeds. The wide range of traits and breeds studied, along with the genotypic information on polymorphisms previously associated with different lipid traits, provide a broad characterization of beef meat, which allows giving a better response to the variety of consumers’ preferences. Also, the development and implementation of low-density SNP panels with predictive value for economically important traits, such as those summarized here, may be used to improve production efficiency and meat quality in the beef industry.

## Background

Cattle meat provides several nutrients necessary for a balanced diet and for health preservation, especially high value proteins, minerals, B-complex vitamins and essential fatty acids (FA), and also can have an important role as a dietary source of n-3 FA and conjugated linoleic acids (CLA) [1,2]. A number of epidemiological studies have associated red meat consumption with increased disease risks [3-5], whereas other authors point out the beneficial effects of the moderate consumption of unprocessed red meat -lowers total cholesterol, LDL cholesterol and triglycerides (TG) [6,7], as well as blood pressure [8]. However, the isolation of the effects of red meat alone is difficult to accomplish [2]. Moreover, the level of intramuscular fat content and FA composition are among the main factors determining meat palatability and consumers satisfaction [9]. Muscle lipid characteristics determine meat flavour and lipid oxidation, which contributes to beef colour, and can be responsible for abnormal odours, and influences the juiciness and tenderness of meat [10]. However, meat quality traits are very complex, involve many genes and are greatly influenced by a variety of environmental factors, such as diet sex, season, age, etc. [11]. Being difficult and expensive to measure [12], they are usually not included in selection programs based on phenotypic performance, and challenge application of traditional selection methods, as well as the state-of-the-art Genomic Selection (GS) [13].

An alternative approach is to identify genes with an effect on fat composition and include these in selection objectives. Thanks to the genomic revolution of the past few years, more information and technology are available that can be used to improve meat quality. Many studies have identified QTL involved in meat quality related traits in beef cattle (e.g. [14,15]); however, the dissection of these QTL has not identified genetic variants explaining a large portion of phenotypic variance [16]. More recently, single nucleotide polymorphisms (SNP) within candidate genes have been tested for predictive value for carcass traits, and some commercial tests to genotype animals based on SNP marker panels are being proposed to breeders (see the review in [17]). The significant progress made in characterizing changes in tissue FA composition to diet, feeding system and genotype, highlights the potential for further progress to be made through genomic or marker-assisted selection in livestock and the formulation of diets to exploit the genetic potential [18]. Nevertheless, the full development of these technologies greatly depends on the precise identification of the genes and polymorphisms that have a measurable effect on muscle physiology and on meat quality, and the validation of their effects on different breeds.

In this study, we merged phenotypic and genotypic information [19-21] to characterize the variation in lipid profile and sensory parameters of *Longissimus thoracis* muscle and to represent the diversity among 15 cattle populations, reared under comparable management conditions. Correlations between fat content, organoleptic characteristics and lipid profiles were also investigated.

## Methods

### Animals and feed system

A total of 436 muscle samples from unrelated bulls belonging to 15 European cattle breeds were studied in the frame of the GeMQual (EC QLK5 – CT2000-0147) European project and genotyped. The breeds included specialized beef breeds, dairy breeds, and local beef breeds. The whole sample included 31 Jersey (JER), 27 South Devon (SD), 30 Aberdeen Angus (AA), and 29 Highland (HIG) from United Kingdom; 29 Holstein (HOL), 29 Danish Red (DR), and 20 Simmental (SM), from Denmark; 30 Asturiana de los Valles (AST), 31 Asturiana de la Montaña (CAS), 30 Avileña-Negra Ibérica (AVI), and 31 Pirenaica (PI) from Spain; 30 Piedmontese (PIE), and 28 Marchigiana (MAR) from Italy; and 31 Limousin (LIM), and 30 Charolais (CHA) from France.

Bulls were reared in each country in a unique location and under a uniform beef management system representative of those used in the European Union (EU) countries. Feed composition and management details are described in [22]. The diet was designed to achieve the slaughter weight of 75% of mature weight for each breed within a window of 13 to 17 months. Animals from each breed were slaughtered the same day in either commercial or experimental abattoirs, depending on the experimental facilities of each country.

### Sampling and phenotype measurements

Carcass processing after slaughter was described by [22] and [23]. For lipid measurements, *Longissimus thoracis* muscle was excised at 24 h postmortem from the left side of the carcass between the 6th and the 13th rib and a sample was taken immediately and frozen for chemical analysis including fat concentration. The remainder was stored at +2°C ± 1°C until 48 h post-mortem. Also, samples were taken from the 48 h post-mortem section to determine total lipid content. Samples for individual FA analysis were taken from the same position on *Longissimus thoracis* from all animals and vacuum packed, frozen and transported on dry ice to University of Bristol (United Kingdom) to determine total lipid content.

Fatness score (FS) corresponds to the visual fatness cover estimated by UE standard (1 = very low, 5 = very high), and fat percentage (FP) is the proportion of subcutaneous and intermuscular fat in the rib dissection. Fat was extracted by the method of [24] separated into neutral lipid and phospholipid, methylated, separated by gas-liquid chromatography (GLC) and the individual peaks identified and quantified as described in detail by [25]. Total lipid content was taken as the sum of the neutral lipid (NL) and phospholipid (PL) fractions. Some additional phenotypes were set as are saturated FA (SFA), monounsaturated FA (MUFA), polyunsaturated FA (PUFA), n-6/n-3 ratios, polyunsaturated to saturated FA (P:S) ratios and antithrombotic potential (ATT), which is the ratio between the sum of the antithrombogenic FAs eicosatrienoic acid (20:3 n-6) and eicosapentaenoic acid (20:5 n-3), and the thrombogenic FA arachidonic acid (20:4 n-6) [(20:3 n-6 + 20:5 n-3)/20:4 n-6] [26]. Sensory panel tests assessed meat using an eight-point scale as described in [27]. The criteria assessed were flavour, texture and juiciness -the higher scores corresponding to the characteristic flavour of beef, and very tender and juicy meat, respectively. See [21] for detailed phenotype values per breed.

### Marker selection

The allele frequencies of 11 polymorphisms found to be associated with different lipid traits across breeds and causing increases in traits ranging between 3.3% and 19% for one homozygous genotype compared to the other homozygous genotype (Table 1) [19,20], were used for a principal components analysis (PCA) to represent the diversity among the 15 cattle populations: calpastatin (*CAST*) g.2959G < A [20]; cofilin 1 (*CFL1*) ss77831721 [19]; EP300 interacting inhibitor of differentiation 1 (*CRI1*) ss778332128 [19]; myostatin (*GDF8*) ss77831865 [20]; insulin-like growth factor 2 receptor (*IGF2R*) ss77831885 [19]; lipoprotein lipase (*LPL*) ss65478732 [20]; matrix metalloproteinase 1 (*MMP1*) ss77831916, ss77831924 [19]; myozenin 1 (*MYOZ1*) ss77831945 [20]; phospholipid transfer protein (PLTP) ss77832104 [19]; peroxisome proliferator activated receptor γ (*PPARG*) ss62850198 [20]. The six SNPs from [19] were genotyped by Kbioscience using the proprietary Kaspar© methodology; the five SNPs from [20] were genotyped by SNP multiplex and Primer Extension amplification.

**Table 1:** **Allele frequencies per breed of 11 polymorphisms with effects ranging between 3.3% and 19% on different lipid traits based on results from **[19,20]

**Locus symbol**	**dbSNP**^**1**^	**Alleles**^**2**^	**Effect of the homozygous genotype for the bold allele**	**Frequency of bold allele**														
				**JER**^**3 **^**(n = 30)**	**SD**^**3 **^**(n = 27)**	**AA**^**3 **^**(n = 30)**	**HIG**^**3 **^**(n = 29)**	**HOL**^**3 **^**(n = 29)**	**DR**^**3 **^**(n = 29)**	**SM**^**3 **^**(n = 20)**	**LIM**^**3 **^**(n = 31)**	**CHA**^**3 **^**(n = 31)**	**PIE**^**3 **^**(n = 30)**	**MAR**^**3 **^**(n = 28)**	**AST**^**3 **^**(n = 30)**	**CAS**^**3 **^**(n = 31)**	**AVI**^**3 **^**(n = 30)**	**PI**^**3 **^**(n = 31)**
CAST^*^	g.2959G < A	**A**/G	↑ 5% FS^4^	0.77	0.83	0.88	0.94	0.69	0.66	0.78	0.68	0.65	0.7	0.64	0.73	0.84	0.85	0.65
CFL1^*^	ss77831721	**C**/T	↑ 8% 18:2/18:3	0.48	0.02	0.12	0.09	0.14	0.43	0.29	0.47	0.61	0.47	0.59	0.39	0.29	0.14	0.22
CRI1^*^	ss77832128	**G**/T	↑ 13.4% N 22:4n-6^5^	0.95	1	1	0.67	0.75	1	0.75	0.9	0.91	0.75	0.92	0.96	0.89	0.72	0.9
GDF8^*^	ss77831865	**G**/del	↑ 15% FS	1	0.65	1	1	1	1	1	1	1	1	1	0.32	0.98	1	0.89
IGF2R^*^	ss77831885	A/**G**	↑ 4.4% Flavour	0.04	0.44	0.32	0.52	0.06	0.33	0.3	0.18	0.44	0.18	0.09	0.13	0.1	0.2	0.22
LPL^*^	ss65478732	**T**/C	↑ N n-6^6^	0	0	0.06	0	0.1	0.05	0.06	0	0.1	0.05	0	0	0.08	0.09	0.02
MMP1^*^	ss77831916	**A**/G	↑ 3.3% CLA	1	0.95	0.79	0.76	0.82	0.85	1	0.97	0.95	0.95	0.74	0.85	0.98	0.91	0.91
***MMP1b***	ss77831924	T/**C**	↑ 14% 22:6n-3	0.5	0.37	0.55	0.44	0.5	0.5	0.5	0.5	0.48	0.47	0.54	0.47	0.5	0.52	0.5
MYOZ1^*^	ss77831945	C/**T**	↑ 8% 18:2/18:3	0.48	0.94	1	0.44	0.63	0.83	0.91	0.8	0.91	0.87	0.82	0.83	0.92	0.92	0.94
PLTP^*^	ss77832104	**G**/A	↑ 8% n-6/n-3	0.5	0.33	0.58	0.25	0.27	0.2	0.38	0.71	0.4	0.63	0.7	0.57	0.08	0.56	0.5
PPARG^*^	ss62850198	G/**A**	↑ n-3^7^	0.13	0.21	0.28	0.04	0.12	0.19	0.14	0.18	0.25	0.12	0.11	0.1	0.1	0.06	0.16

*Genes for which allele frequencies are statistically different among breeds (*P <* 0.0001).

^1^dbSNPs accession number of SNPs found to be associated with different production traits by [19,20].

^2^In bold, favourable allele, i.e. allele which improves animal performance and/or meat quality by diminishing n-6 and/or n-6:n-3 ratio, by increasing n-3, or by improving organoleptic characteristics.

^3^Complete breed names: Jersey (JER), South Devon (SD), Aberdeen Angus (AA), Highland (HIG), Holstein (HOL), Danish Red (DR), Simmental (SM), Limousin (LIM), Charolais (CHA), Piedmontese (PIE), Marchigiana (MAR), Asturiana de los Valles (AST), Asturiana de la Montaña (CAS), Avileña-Negra Ibérica (AVI), Pirenaica (PI). Between brackets: maximum n.

^4^Fatness score.

^5^N: neutral fatty acid.

^6^↑16% neutral 20:3 n-6; ↑ 19% neutral 20:4 n-6.

^7^↑ 9% 22:5 n-3; ↑ 15% 20:5 n-3; ↑ 18% 22:6 n-3.

### Statistical analysis

Spearman correlations were determined between fatness score, fat percentage, flavour, juiciness, texture, and different lipid profiles of *Longissimus thoracis* muscle in 15 European cattle breeds using the CORR procedure of SAS and considering the whole set of data on all animals. Allele frequency data were subjected to ANOVA, using the PROC GLM procedure of SAS and considering breed as independent variable. A PCA procedure was performed using the mean of phenotypic measurements by breed and allelic frequencies from 11 polymorphisms to determine the main traits and SNPs that explained most of the variation among the 15 cattle populations. The frequency of the allele showing a positive correlation with the trait was used (bold allele in Table 1). All these statistical analyses were carried out using the SAS statistical package v. 9.1.3 [28].

## Results

### Trait correlations

Table 2 shows the main correlations between FS, FP, flavour, juiciness, texture, and different lipid profiles of *Longissimus thoracis*. FS correlated positively with FP (r = 0.62, P < 0.001), and both of them with absolute amounts of lipids in muscle, including total PL and NL, SFA, MUFA, PUFA, n-3 and n-6 content, as well as with flavour score. However, both of them displayed a negative correlation to P:S ratios -explained by their higher correlations with SFA content (FS r = 0.4, P < 0.001; FP r = 0.68, P < 0.001) than to PUFA (FS r = 0.17, P < 0.001; FP r = 0.44, P < 0.001)-, and to n-6/n-3 ratios -because of their lower correlations to n-6 (FS r = 0.13, P < 0.01; FP r = 0.37, P < 0.001) compared to n-3 (FS r = 0.28, P < 0.001; FP r = 0.56, P < 0.001)-. The correlation between total PL and FS (r = 0.11, P < 0.05) and FP (r = 0.44, P < 0.001) is lower than between total NL and these traits (FS r = 0.44, P < 0.001; FP r = 0.78, P < 0.001). Similar correlation coefficients were also observed between PL and total lipid (r = 0.7, P < 0.001) and SFA (r = 0.66, P < 0.001), compared to NL with both total lipid and SFA (r = 0.99, P < 0.001). The correlation between SFA and MUFA (r = 0.99, P < 0.001) was higher than between SFA and PUFA (r = 0.7, P < 0.001). The proportion of 18:2 n-6 declines in muscle as fat deposition increases (correlations with FP r = −0.77, P < 0.001, total lipid r = −0.86, P < 0.001, and SFA r = −0.88, P < 0.001).

**Table 2:** **Correlations between fatness score, fat percentage, flavour, juiciness, texture, and different lipid profiles of *Longissimus thoracis *** **muscle in 15 European cattle breeds**

	**FS**^**1**^	**FP**^**2**^	**Flavour**	**Juiciness**	**Texture**	**Total lipid**	**SFA**
FP	0.62***						
Flavour	0.12*	0.31***					
Juiciness	0.07	0.01	0.21***				
Texture	0.06	0.01	0.16***	0.57***			
Total Lipid	0.43***	0.77***	0.3***	−0.04	−0.02		
SFA^3^	0.4***	0.68***	0.3***	−0.01	0.06	0.95***	
MUFA^4^	0.41***	0.67***	0.28***	−0.02	0.06	0.99***	0.99***
PUFA^5^	0.17***	0.44***	0.27***	−0.1*	−0.02	0.73***	0.7***
% PUFA	−0.51***	−0.77***	−0.25***	−0.01	−0.02	−0.9***	−0.92***
% 18:2 n-6	−0.53***	−0.77***	−0.25***	−0.01	−0.02	−0.86***	−0.88***
Total PL	0.11*	0.44***	0.26***	−0.13**	−0.06	0.7***	0.66***
Total NL	0.44***	0.78***	0.3***	−0.03	−0.01	0.99***	0.99***
n-3 PUFA^6^	0.28***	0.56***	0.42***	0.07	−0.02	0.63***	0.62***
% n-3PUFA	−0.24***	−0.34***	−0.01	0.08	−0.06	−0.62***	−0.62***
n-6 PUFA^7^	0.13**	0.37***	0.21***	−0.13**	−0.02	0.68***	0.64***
% n-6PUFA	−0.53***	−0.79***	−0.27***	−0.02	−0.02	−0.9***	−0.91***
P:S1^8^	−0.44***	−0.68***	−0.16***	−0.01	−0.02	−0.85***	−0.87***
P:S2^9^	−0.44***	−0.69***	−0.18***	−0.01	−0.01	−0.9***	−0.92***
n-6/n-3	−0.28***	−0.47***	−0.24***	−0.08	0.04	−0.26***	−0.27***
18:2/18:3	−0.38***	−0.67***	−0.28***	−0.02	0.04	−0.6***	−0.61***
22:6/18:3	0.04	−0.29***	−0.26***	−0.06	−0.03	−0.38***	−0.38***
ATT^10^	0.35***	0.44***	0.22***	0.13**	0.01	0.06	0.2***

Level of significance: ***(P < 0.001), **(P < 0.01), *(P < 0.05).

^1^Fatness score: visual fatness cover estimated by UE standard.

^2^Fatness percentage: proportion of fat (subcutaneous and intermuscular) in the rib dissection.

^3^12:0 + 14:0 + 16:0 + 18:0.

^4^16:1 + t18:1 + 9c18:1+ 11c18:1 + 20:1.

^5^18:2n-6 + 18:3n-3 + 20:3n-6 + 20:4n-6 + 20:5n-3 + 22:4n-6 + 22:5n-3 + 22:6n-3.

^6^18:3n-3 + 20:5n-3 + 22:5n-3 + 22:6n-3.

^7^18:2n-6 + 20:3n-6 + 20:4n-6 + 22:4n-6.

^8^(18:2n-6 + 18:3n-3) / (12:0 + 14:0 + 16:0 + 18:0).

^9^(18:2n-6 + 18:3n-3 + 20:3n-6 + 20:4n-6 + 20:5n-3 + 22:4n-6 + 22:5n-3 + 22:6n-3) / (12:0 + 14:0 + 16:0 + 18:0).

^10^(20:3n-6 + 20:5n-3)/20:4n-6.

Flavour correlated positively with the organoleptic characteristics juiciness (r = 0.21, P < 0.001) and texture (r = 0.16, P < 0.001). A higher positive correlation was found between juiciness and texture (r = 0.57, P < 0.001). Finally, beef juiciness showed a small negative correlation to the amount of PL (r = −0.13, P < 0.01) and PUFA (r = −0.1, P < 0.05), particularly to n-6 content (r = −0.13, P < 0.01).

Apart from results in Table 2, it is worth highlighting the positive correlation between 18:1 trans-vaccenic FA (t18:1) and CLA cis-9,trans-11 (r = 0.62, P < 0.001).

### Phenotype and genotype variation among breeds

**Figure 1:**
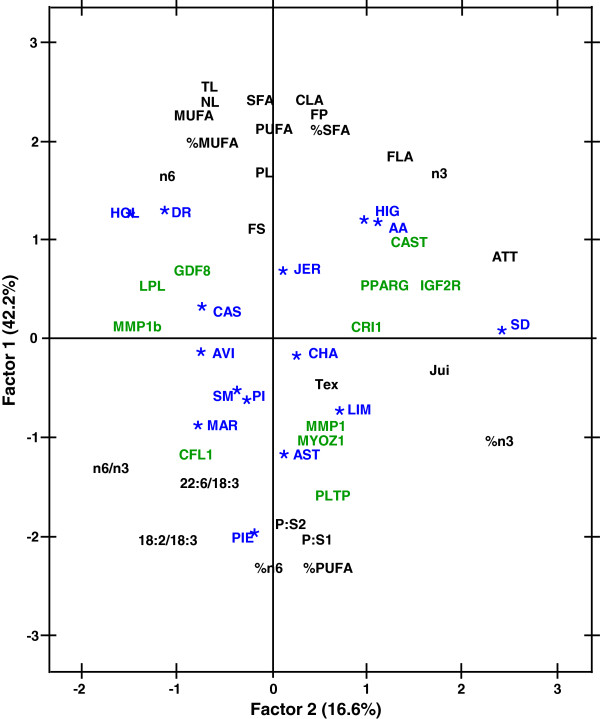
**Plot of factor pattern for factors 1 and 2 of 15 European breeds showing the correlations to lipid traits (in black) and genotypic data (in green) from 11 polymorphisms with the two principal components.** Abbreviations: TL total lipids, NL neutral lipids, PL phospholipids, FP fatness percentage, FS fatness score, Fla flavour, Tex texture, Jui juiciness, JER Jersey, SD South Devon, AA Aberdeen Angus, HIG Highland, HOL Holstein, DR Danish Red, SM Simmental, LIM Limousin, CHA Charolais, PIE Piedmontese, MAR Marchigiana, AST Asturiana de los Valles, CAS Asturiana de la Montaña, AVI Avileña-Negra Ibérica, PI Pirenaica.

The plot of factor pattern of the 15 cattle breeds showing the correlations to lipid traits and genotypic data from 11 polymorphisms with the two principal components is shown in Figure 1. The first two dimensions (Factor 1, 42.2%; Factor 2, 16.6%) explained 58.8% of the variation among breeds (Figure 1). When considering the different lipid traits, the first dimension was mainly influenced by total lipid measurements and flavour score, whereas on the opposite side muscle percentages of PUFA and of n-6, as well as P:S1, P:S2 and 18:2/18:3 ratios are plotted. The second dimension was mainly influenced by the ATT index, n-3, % n-3, and juiciness. Therefore, AA, HIG, HOL, DR and JER breeds, which displayed higher fatness [21], appeared in the positive area of the first dimension and split into two groups by the influence of the higher n-3 muscle content and flavour scores of AA and HIG breeds, and by the higher n-6 and MUFA content of HOL and DR dairy breeds. In contrast, lean breeds with high proportion of PUFAs, and high P:S and n-6 to n-3 ratios, such as PIE and AST [21], appeared at the bottom of the plot (Figure 1). Finally, SD breed stands out because of its highest ATT ratio and percentage of n-3 in muscle (Figure 1).

Concerning SNPs distribution, they plotted mainly according to their previous trait associations, although also influenced by their allele frequencies per breed (Table 1).

## Discussion

All animals included in this project (GeMQual QLK5 – CT2000-0147) were fed a similar diet and reared intensively under comparable management conditions between countries. The effects of all factors other than breed (country, diet, slaughter) were controlled to minimize differences and were confounded with the breed effect. Inevitably, some variations might have occurred but special emphasis has been put to respect the diet composition in the different countries. The higher absolute n-3 PUFA muscle content found in the UK breeds, especially in the Aberdeen Angus breed, cannot be due to a grass-based diet generally used in UK [29] inexistent in this study, but rather to a specific characteristic of this fat breed.

### Trait correlations

The correlation coefficients between total muscle lipid measurements (FS, FP, total lipids, SFA), and absolute vs. proportional (%) amounts of PUFA, n-6 and n-3 FAs were in opposite directions [30,31]. For example, the sum of n-3 FA showed a positive relation to FS (r = 0.28), FP (r = 0.56), total lipids (r = 0.63) and SFA (r = 0.62) for absolute amounts, but there were negative correlations between those traits and n-3 relative proportion (FS r = −0.24, FP r = −0.34, total lipids r = −0.62, SFA r = −0.62) (Table 2). In particular, the negative correlation obtained here between the percentage of 18:2 n-6 and fat measurements, as well as the weaker correlation between total PL and FS, FP, total lipid and SFA muscle content compared with NL (Table 2) [32], is in accordance with the expected proportions of PL vs. NL as animal fattens. Long chain PUFAs are mainly stored in muscle PL in cattle, which is an essential component of cell membranes and its amount remains fairly constant as the animal fattens, whereas NL increases in overall FA composition. SFA and MUFA are mainly stored in the NL fraction in triglycerides. This means increasing carcass fat leads to an increasing amount of FAs in triglycerides, but at the same time the relative amount of PUFAs is decreasing [32], which is in concordance with the negative correlation obtained here between the percentage of PUFA and fat measurements, as well as the weaker correlation between total PL and total lipid (r = 0.7) muscle content compared with NL (r = 0.99) (Table 2).

In agreement also with previous studies [33,34], we found a positive correlation between t18:1 and CLA (r = 0.62, *P* < 0.001), explained by the metabolic relationships between both FA –in ruminants the SCD enzyme forms also CLA from t18:1 in adipose tissue [29,32].

Although high levels of long chain n-3 PUFA have been described as having an impact on flavour to produce a ‘grass fed’ taste [35,36], and [37] found no correlation between n-6 PUFA and flavour in two beef breeds, here there was only a negative correlation to the percentage of total PUFA, particularly to n-6 fraction, whereas the percentage of n-3 in muscle did not seem to influence meat flavour in cattle not fed with a grass-based diet.

As expected, the correlation between juiciness and texture is higher than with flavour scores given that juiciness depends mainly on the meat water-binding capacity and plays a key role in meat texture [38-40], contributing to its variability [41], whereas flavour is mainly influenced by FA composition and marbling [42], as reflected by its positive correlations with all absolute fat content measurements obtained here (Table 2). Although texture and juiciness properties also are dependent on other characteristics of meat, including fat content [40], both of them showed few or no correlation with muscle fat content or FA profile (Table 2).

### Phenotype and genotype variation among breeds

The distribution of breeds plotted by PCA analysis fell into three main groups (Figure 1): one group defined as having a high absolute fat content, which splits into two blocks –AA and HIG breeds on one hand, characterized by higher n-3 muscle content and flavour scores, and in the other hand HOL and DR dairy breeds, which displayed higher values of MUFA and n-6-; a second group with lower fat content and higher proportion of PUFAs, as well as PUFA vs. SFA ratios (healthier meat) -PIE and AST-; and a large group gathering the rest of breeds with intermediate fat content, among which it is worth highlighting SD because of its highest ATT ratio (index higher values better for health) and percentage of n-3 in muscle.

Regarding SNPs distribution, most of them plotted according to their previous trait associations and also influenced by their allele frequencies per breed (Table 1, Figure 1): *PPARG*, which influences the amount of 22:5 n-3, 20:5 n-3 and 22:6 n-3 in muscle [20], appeared near n-3 and ATT ratio -calculated as (20:3 n-6 + 20:5 n-3)/20:4 n-6- factor patterns, as well as almost equidistant to the three breeds with higher allele frequencies for the A allele –AA, CHA and SD-; *CFL1*, *PLTP* and *MYOZ1*, which were associated with n-6 to n-3 ratio [19], correlated with 18:2 n-6/18:3 n-3 and specially with n-6/n-3 ratio in Factor 1; *IGF2R,* previously linked to an increase in flavour [19], shared Factor 2 pattern with flavour and was placed almost equidistant to the three breeds with higher allele frequencies for the G allele –HIG, CHA and SD-; *CAST* was associated with an increase in FS and appeared closely related to the two breeds with higher allele frequencies for the A allele –AA and HIG-, sharing also Factor 1 pattern with FS; *LPL*, associated with the increase of several neutral n-6 [20], plotted in the same Factor 2 pattern than n-6 content, but closer to and almost equidistant from the three breeds with higher allele frequencies for the T allele –HOL, AVI and CAS-; and, as expected, *GDF8* SNP was placed near the trait FS [20].

Finally, there were no relationships between the two SNPs in the *MMP1* gene and the SNP in *CRI1* neither with their main trait associations –CLA, 22:6 n-3 and 22:4 n-6, respectively [19]-, nor with breed allele frequencies, which may be caused by the other trait associations of this SNPs with lower or unknown effects [19].

## Conclusions

The wide range of traits and breeds studied, along with the genotypic information on polymorphisms previously associated with different lipid traits, provide a broad characterization of the phenotypic and genotypic background underlying variations in FA composition and sensory parameters between breeds, which allows giving a better response to the variety of consumers’ preferences. Also, the development and implementation of low-density SNP panels with predictive value for economically important traits, such as those summarized here, may be used to improve production efficiency and meat quality in the beef industry as a molecular signature of GTTdelGCACCAA for *CAST* (g.2959G < A), *CFL1* (ss77831721), *CRI1* (ss77832128), *GDF8* (ss77831865), *IGF2R* (ss77831885), *LPL* (ss65478732), *MMP1* (ss77831916, ss77831924), *MYOZ1* (ss77831945), *PLTP* (ss77832104), and *PPARG* (ss62850198), respectively, which would correspond to the “most favourable” haplotype.

## Competing interests

The authors declare that they have no competing interests.

## Authors’ contributions

NS carried out the molecular genetic studies, performed the statistical analysis and drafted the manuscript. SD, HL, GN, CS, AV and JLW conceived the study, and participated in its design and coordination. SD also helped to draft the manuscript. All authors read and approved the final manuscript.
